# Qualitative Prediction of Ligand Dissociation Kinetics from Focal Adhesion Kinase Using Steered Molecular Dynamics

**DOI:** 10.3390/life11020074

**Published:** 2021-01-20

**Authors:** Justin Spiriti, Chung F. Wong

**Affiliations:** Department of Chemistry and Bochemistry, University of Missouri-St. Louis, St. Louis, MO 63121-4400, USA; spiritij@umsl.edu

**Keywords:** focal adhesion kinase, drug-binding kinetics, free energy methods, umbrella sampling, steered molecular dynamics

## Abstract

Most early-stage drug discovery projects focus on equilibrium binding affinity to the target alongside selectivity and other pharmaceutical properties. Since many approved drugs have nonequilibrium binding characteristics, there has been increasing interest in optimizing binding kinetics early in the drug discovery process. As focal adhesion kinase (FAK) is an important drug target, we examine whether steered molecular dynamics (SMD) can be useful for identifying drug candidates with the desired drug-binding kinetics. In simulating the dissociation of 14 ligands from FAK, we find an empirical power–law relationship between the simulated time needed for ligand unbinding and the experimental rate constant for dissociation, with a strong correlation depending on the SMD force used. To improve predictions, we further develop regression models connecting experimental dissociation rate with various structural and energetic quantities derived from the simulations. These models can be used to predict dissociation rates from FAK for related compounds.

## 1. Introduction

Most drugs function by binding to a specific target and altering its activity in a way that ultimately prevents or treats a disease. Consequently, while a wide range of factors affect the clinical efficacy and safety of a drug candidate (including selectivity, toxicity, solubility and pharmacokinetic properties), the binding affinity of a drug candidate to its target is of primary importance, and high binding affinity can make up for less desirable characteristics elsewhere. Accordingly, many drug discovery efforts begin by searching for compounds that have high affinity for binding to the target. Computational methods have become an important part of this effort [1], and a wide range of methods have been developed for determining binding affinities. The most rigorous and computationally expensive methods involve alchemical free energy methods [2,3,4,5,6]. These methods take advantage of the fact that free energy is a state function by effectively causing the ligand to appear within the binding site of the protein or in solvent and determining the free energy changes for these “alchemical’’ transformations. The free energy change for the dissociation of the ligand from the target can then be calculated using a thermodynamic cycle. Other less rigorous methods include MM/PBSA and MM/GBSA methods [7,8], which determine the affinity using energies calculated from simulations with and without the ligand, and molecular docking, which uses scoring functions that have been fitted to correlate with the binding affinity [9,10,11].

There is increasing evidence that the in vivo effectiveness of some drug candidates depends not only on their equilibrium binding affinity but also on their residence time bound to their targets. The advantages of a longer residence time at the target can include better selectivity, a larger therapeutic window and increased duration of action or less frequent dosing [12,13,14], although there is not universal agreement that residence time offers more information than equilibrium binding affinity [15]. Nevertheless, the pharmaceutical industry is making an effort to develop a better understanding of the factors affecting drug residence time. Computational simulations of biomolecules can play an important role in this effort [16,17]. It is more difficult to directly simulate the dissociation of a ligand from a target than to apply free energy techniques to determine the binding affinity because the timescales on which ligands dissociate are usually orders of magnitude longer than those directly accessible to simulation. The binding equilibrium of ligand fragments to FKBP has been directly observed using simulations on the microsecond timescale carried out on the Anton supercomputer [18]. In addition, there are a number of enhanced sampling methods that can determine the rates of long timescale processes from simulations on shorter timescales. These include Markov state analysis of unbiased simulations [19,20,21], the weighted ensemble method [22,23,24,25,26], multiple replica scaled MD [27], selectively scaled MD simulations [28], τ-RAMD simulations [29], milestoning [30,31], combined weighed ensemble and milestoning [32] and transition path sampling [33,34]. Other methods, while not directly yielding estimates of dissociation rates, could be used to obtain pathways that ligands might take in dissociating from the binding site of the target. These include steered molecular dynamics [35,36], targeted molecular dynamics [37,38] and biased molecular dynamics [39], which use additional forces or constraints in different ways to cause conformational changes to take place much faster than they would in an unbiased simulation. Once such a pathway has been identified, it can be used to define the reaction coordinate in more rigorous but time-consuming simulations such as computing free energy profiles or surfaces by umbrella sampling [40].

Combining these methods can achieve accurate predictions of residence times. For example, the dissociation of ligands from the A2A adenosine receptor, a G-protein coupled receptor, has been simulated by using SMD to pull the ligand from the binding site into the extracellular vestibule, and the calculated change in the interaction energy between ligand and water over the course of the simulation was found to have a strong correlation with the experimental dissociation rate [41]. Enhanced sampling techniques can also be used to explore protein conformational changes that are critical to ligand unbinding and thereby improve the accuracy of predicted dissociation rates. For example, combining SMD, infrequent metadynamics, and Markov analysis allowed detailed study of the unbinding mechanism of a radioligand from the α7 nicotinic acetylcholine receptor as well as estimation of the dissociation rate within an order of magnitude of the experimental value [42]. Finally, using methods similar to those used here, the dissociation of eight ligands from the protein kinase p38α was studied by first performing SMD to determine unbinding paths, then resampling again using SMD but in a manner similar to umbrella sampling to determine the potential of mean force for unbinding. The barrier heights from this potential of mean force for each ligand were then used to predict the dissociation rate, and a high correlation of 0.86 with the experimental dissociation rate was observed. However, this method was relatively expensive computationally, requiring a total of 4.5 μs for each ligand [43].

Recently, our group tested the concept of using steered molecular dynamics simulations to predict the dissociation rates of three ligands from focal adhesion kinase (FAK) simulations [44], comparing the results to dissociation rates for these ligands measured using surface plasmon resonance [45]. FAK has attracted interest as a target for the development of anticancer therapies because it plays an important role in mediating interactions between cells and the extracellular matrix, activating pathways that promote cell growth and survival in response to integrin binding to the extracellular matrix [46,47]. The activity of FAK is thought to contribute to the ability of cancer cells to survive, grow, and metastasize even in the absence of attachment to the extracellular matrix [48]. In our previous SMD study, we found that SMD could rank the dissociation rates for the three ligands in the same order as the experimental measurements. Furthermore, dissociation pathways for the ligands appeared to have a two-step mechanism, with an intermediate state and the exposure of a hydrophobic phenyl ring contributes to the activation barrier.

In addition to SMD simulations, we have used Markov state analysis and the milestoning method to estimate the dissociation rate of one of the ligands (Wong, not published) and found the rate to exceed the experimental value by 6–7 orders of magnitude. We then performed umbrella sampling simulations to determine the free energy surface for dissociation and found a low barrier giving a similar overestimate of the dissociation rate (results shown below). Therefore, we turn to SMD simulations again to explore its capability as an inexpensive method to simply classify compounds into fast or slow dissociating ligands. Initially, we simply hoped that the method could increase the enrichment factor in screening compounds with the desired drug-binding kinetics, such as long residence time, as molecular docking could for finding strong binders. This is already useful for practical drug discovery. To our surprise, the study of 14 ligands here suggests that the method can even rank-order the dissociation rate of these ligands, especially by introducing regression models that connect experimental measurements with various energetic or structural parameters from the simulations. The regression models we develop may be useful for predicting the dissociation rate of other ligands from FAK in a computationally inexpensive way.

## 2. Results

### 2.1. Free Energy Surface for Exit of Ligands from the Binding Site

**Figure 1:**
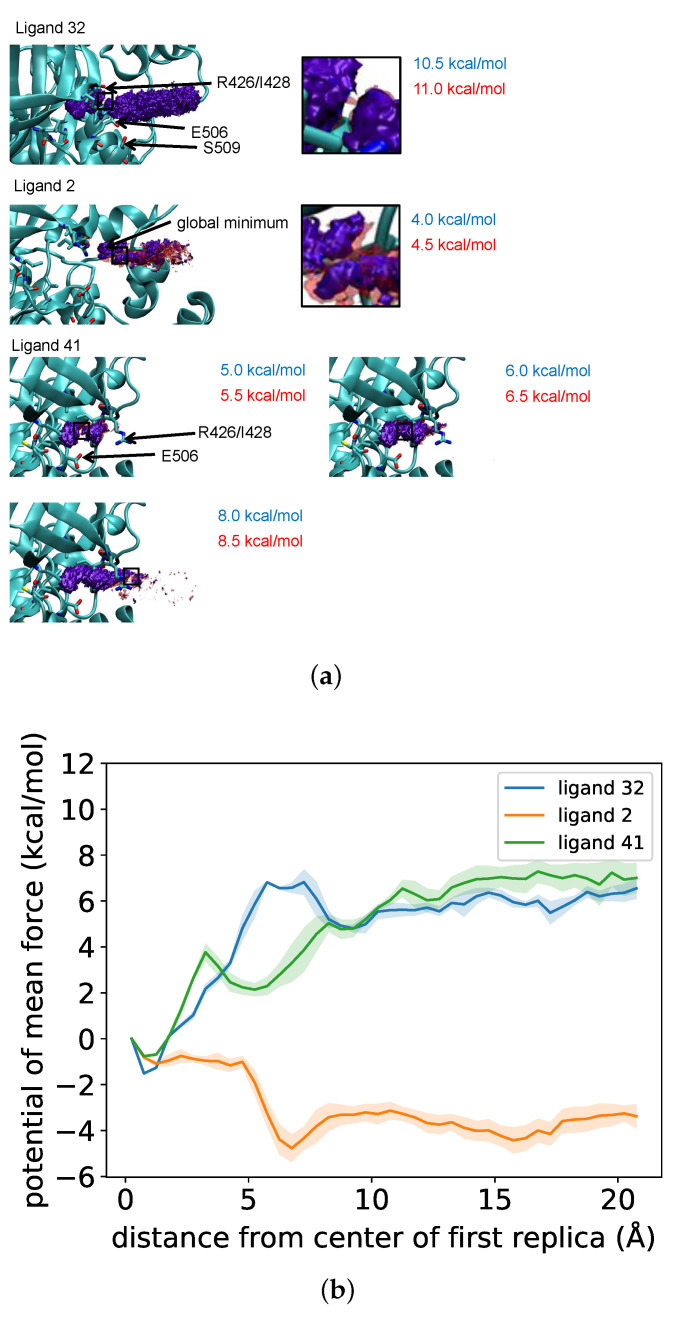
(**a**) Contour plots of the free energy surface of the center of mass of ligands **32**, **2**, and **41** relative to FAK as determined from umbrella sampling simulations. Each image shows two contour surfaces. The blue contour was obtained just below the free energy threshold before two neighboring structures along the reaction coordinate could be connected. The red contour was obtained right after the two structures could be connected. The free energies of the two colored contours thus give us an idea on the energy threshold when the transition state started to form. Three images are shown for ligand **41** because its more stable intermediate state gives a somewhat different picture. A transition state can be seen that forms between the blue contour at 5.0 kcal/mol and the red contour at 5.5 kcal/mol. However, because the barrier for dissociation from the intermediate state to the unbound state is large, this contour does not extend towards the unbound state. The contours at 6.0–6.5 kcal/mol and 8.0–8.5 kcal/mol show the sequence as the unbound state is reached at higher free energies. For ligands **32** and **2**, the insets show close-up views when the transition states begin to form. (**b**) One-dimensional potential of mean force as a function of the distance of the center of mass of the ligand from the binding site.

We first examined the free energy surface for the dissociation of ligands **32**, **2**, and **41** from the active site of FAK to get a sense for the mechanism of dissociation and the location and nature of the activation barrier. Figure 1 shows the three-dimensional free energy surface for the center of mass of ligand **32** relative to FAK as a series of contour surfaces. Figure 1b shows the one-dimensional potential of mean force along the dissociation path, which has been obtained from the three-dimensional free energy surface by integrating out two of the degrees of freedom. Since only conformations in which the ligand was near the exit pathway previously identified by SMD were sampled, only the free energy near this pathway was well estimated. The profile of the potential of mean force shows a free energy basin corresponding to the bound state and a shallow basin for an intermediate state before ligand dissociation. This qualitatively agrees with the two-step dissociation mechanism observed in previous SMD simulations carried out in explicit solvent [44]. The free energy surface shows the dissociation pathway in the structure of the protein. Figure 1 displays the free energy contours just before the bound state and intermediate state can be connected and after—10.5 kcal/mol and 11.0 kcal/mol, respectively. It illustrates the location of the center of mass of the ligand at the transition state. It is located near the side chains of Arg 426, Glu 506, and Ser 509.

If one estimates the activation barrier for the dissociation of ligand 32 by the average of 10.5 kcal/mol and 11.0 kcal/mol and uses it to estimate the order of magnitude of the dissociation rate by Eyring’s transition state equation [49,50].

(1) $$k_{d}=\frac{\kappa k_{B}T}{h}\exp\left[-\frac{\Delta G^{‡}}{k_{B}T}\right]$$

where $\Delta G^{‡}$ is the transition state free energy, *T* is the temperature, and κ the transmission coefficient (the fraction of reactions that proceed to products after reaching the transition state, which we assume to be 1). This corresponds to a dissociation rate of approximately $2\times 10^{5}\;s^{-1}$, some seven orders of magnitude larger than the experimental dissociation rate for ligand **32**. Similar results were obtained in previous attempts to calculate ligand dissociation rates using unbiased MD simulations and Markov chain analysis (Wong, unpublished). If one uses the activation barrier from the potential of mean force, which is smaller at about 8 kcal/mol, the dissociation rate deviates further from the experimental value.

The profile of the one-dimensional potential of mean force (PMF) for ligand **2** is quite different from that of **32**. The global minimum corresponds to a bound structure in which the center of mass of ligand **2** is approximately 6 Å away from the position shown in the crystal structure of ligand **32**. The height of the dissociation barrier is approximately 4–4.5 kcal/mol above this global minimum. (Because of the averaging involved in going from a three-dimensional free energy surface to a one-dimensional PMF, this barrier appears to be only approximately 2 kcal/mol in the one-dimensional PMF.) This is qualitatively consistent with the increased dissociation rate for ligand **2** compared to **32** but may exaggerate it quantitatively. The potential of mean force for ligand **41** appears similar to that of **32**, with a stable bound state and an intermediate state. The free energy difference between the bound and unbound states are similar for the two compounds, which is consistent with them having comparable binding affinities to FAK. However, the energy barriers differ. The barrier for transition from the bound state to the intermediate state is smaller than that of ligand **32**, about 5 kcal/mol. The transition from the intermediate state to the unbound state is about the same, approximately 4 kcal/mol. The free energy surface shows that the bound and intermediate states start to connect with each other between the contours at 5.0 kcal/mol and that at 5.5 kcal/mol. Between 6.0 kcal/mol and 6.5 kcal/mol, the intermediate state starts to connect with a dissociated state. Between 8.0 kcal/mol and 8.5 kcal/mol, the ligand dissociates further out of the protein. The highest barrier height from the potential of mean force appears to be about 5 kcal/mol, which is lower than the barrier height for ligand **32**, despite **41** dissociating more slowly.

### 2.2. Prediction of Experimental Dissociation Rates from Simulated Exit Times

**Figure 2:**
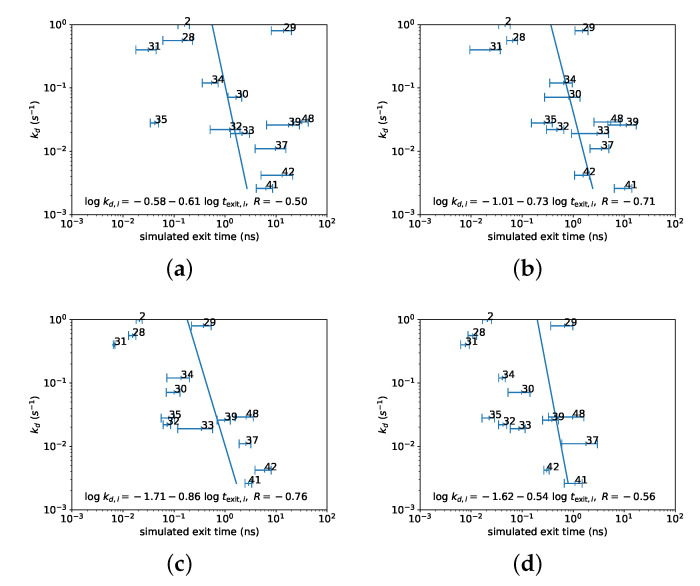
Simulated exit times in steered molecular dynamics (SMD) simulations correlate strongly with experimental dissociation rates. Plots of simulated exit time versus experimental dissociation rate, together with power–law regression according to Equation 2, for simulations conducted with a force of (**a**) 350 pN; (**b**) 400 pN; (**c**) 450 pN; (**d**) 500 pN. All regressions include ligand **29**, which was found to be an outlier (see Table 1).

The SMD simulations reported here used a force to push the ligands out of the binding site, and consequently, the ligands exited the binding site on a much shorter timescale in the simulations compared to experiments. Nevertheless, the time needed to exit the binding site in the simulations was a natural choice for prediction of the experimental dissociation rate. Figure 2 shows the correlation between the exit time as measured in the SMD simulations and the experimental dissociation rates. The data was fitted to the empirical power–law relationship

(2) $$\log_{10}k_{d,i}=\beta_{0}+\beta_{1}\log{\langle t_{\mathrm{exit}}\rangle }_{i}+\varepsilon_{i},\;\varepsilon_{i}∼N\left(0,\sigma^{2}\right)$$

where $k_{d,i}$ is the experimental dissociation rate for compound *i*, and ${\langle t_{\mathrm{exit}}\rangle }_{i}$ is the average of the exit times over the three SMD simulations carried out for compound *i*. (The experimental dissociation rate for compound **2** was very high and outside the instrument range [45]. For the regressions, the dissociation rate was assumed to be 1 s${}^{-1}$, which was the minimum dissociation rate consistent with the measurement).

A strong correlation between simulated exit time and experimental dissociation rate was observed for all four forces used for SMD simulations; a force of 450 pN gave the highest correlation. The values and standard errors for the exponent $\beta_{1}$ and the correlation coefficients are shown in Table 1. The root mean squared deviation of the residuals $\varepsilon_{i}$ was approximately 0.5–0.7 $\log_{10}$ units, which implies that the experimental dissociation rate can be predicted within a factor of approximately 4–5. Despite the strong correlation, the exit time could vary by an order of magnitude for individual SMD simulations with the same ligand. Ligand **29** appeared to be an outlier; the correlations were much higher and the root mean squared error lower when this ligand was removed. Nevertheless, these correlations are encouraging, considering the approximations employed by the SMD simulations to substantially reduce simulation time.

### 2.3. Prediction of Experimental Dissociation Rates from Structural and Energetic Variables

Given the wide range over which simulated exit times were found to vary for each compound, predictive models were also constructed using other independent variables that could be calculated from the simulations. For these regressions, the simulations with an applied force of 400 pN force were used. While the correlation of simulated exit time with experimental dissociation rate was slightly less for an applied force of 400 pN than for a force of 450 pN, we chose to analyze the 400 pN trajectories because they are longer and provide more structural data than the 450 pN trajectories.

The independent variables for which predictive models were constructed included the exposed solvent-accessible surface area of the ligand and the total surface area buried between the protein and ligand, as well as the total interaction energy between ligand and protein (determined using FACTS) and its components. The following model was fitted using the following regression to relate the experimental dissociation rate $k_{d,i}$ to an independent variable $x_{i}$.

(3) $$\log_{10}k_{d,i}=\beta_{0}+\beta_{1}{\langle x\rangle }_{i}+\varepsilon_{i},\;\varepsilon_{i}∼N\left(0,\sigma^{2}\right)$$

**Figure 3:**
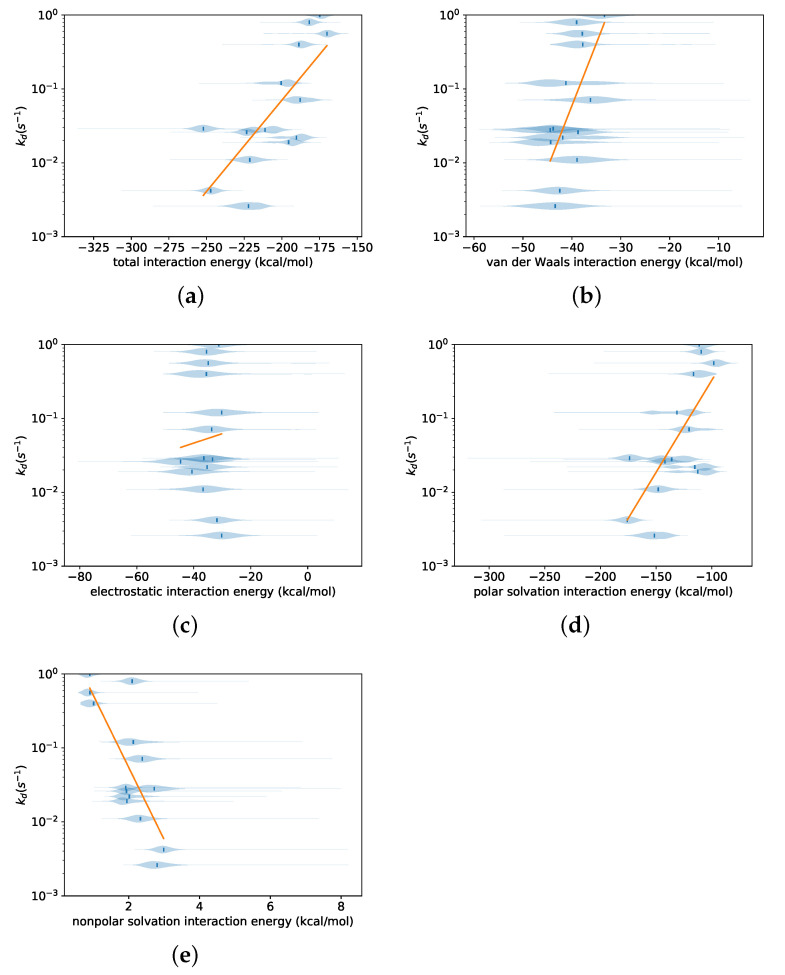
Distribution of total interaction energy between the ligand and protein, and its components, over all three simulations performed for each ligand for a 400 pN force. Regression fits of Equation (3) relating the experimental dissociation rate to the mean values of each distribution are also shown; the slope and standard error of each regression line, Pearson correlation, and *p*-value are shown in Table 2. (**a**) for the total interaction energy; (**b**) for the van der Waals component; (**c**) for the electrostatic component; (**d**) for the polar solvation component; (**e**) for the nonpolar solvation component.

**Figure 4:**
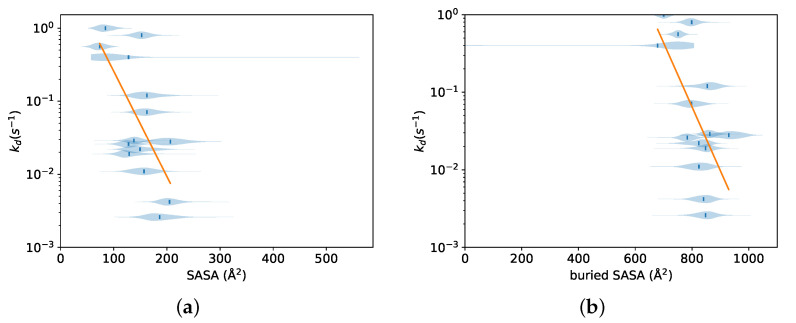
Distribution of ligand or buried solvent accessible surface areas over all three simulations performed for each ligand for a 400 pN force. Regression fits of Equation (3) relating the experimental dissociation rate to the mean values of each distribution are also shown; the slope and standard error of each regression line, Pearson correlation, and *p*-value are shown in Table 2. (**a**) SASA of the ligand in the context of the entire system; (**b**) total buried SASA.

Here, ${\langle x\rangle }_{i}$ is the mean value of either one of the interaction energy terms or one of the surface area terms, calculated over all three simulations for each ligand *i*. Figure 3 shows these regressions for the total interaction energy and its components. The total interaction energy showed a strong correlation to the experimental dissociation rate, slightly greater in absolute value than the simulated exit time for the same force (0.76 vs. 0.71). All of its components except the electrostatic interaction energy also showed strong correlations with the experimental dissociation rate; the strongest was for the nonpolar solvation component, at −0.79. Since the nonpolar solvation component of the FACTS energy function is proportional to the surface area [51], similar regressions were carried out using various surface areas. Figure 4 shows regressions for the ligand surface area and total buried surface area. These also featured strong correlations, although not as high as those for exit time or interaction energy terms.

**Figure 5:**
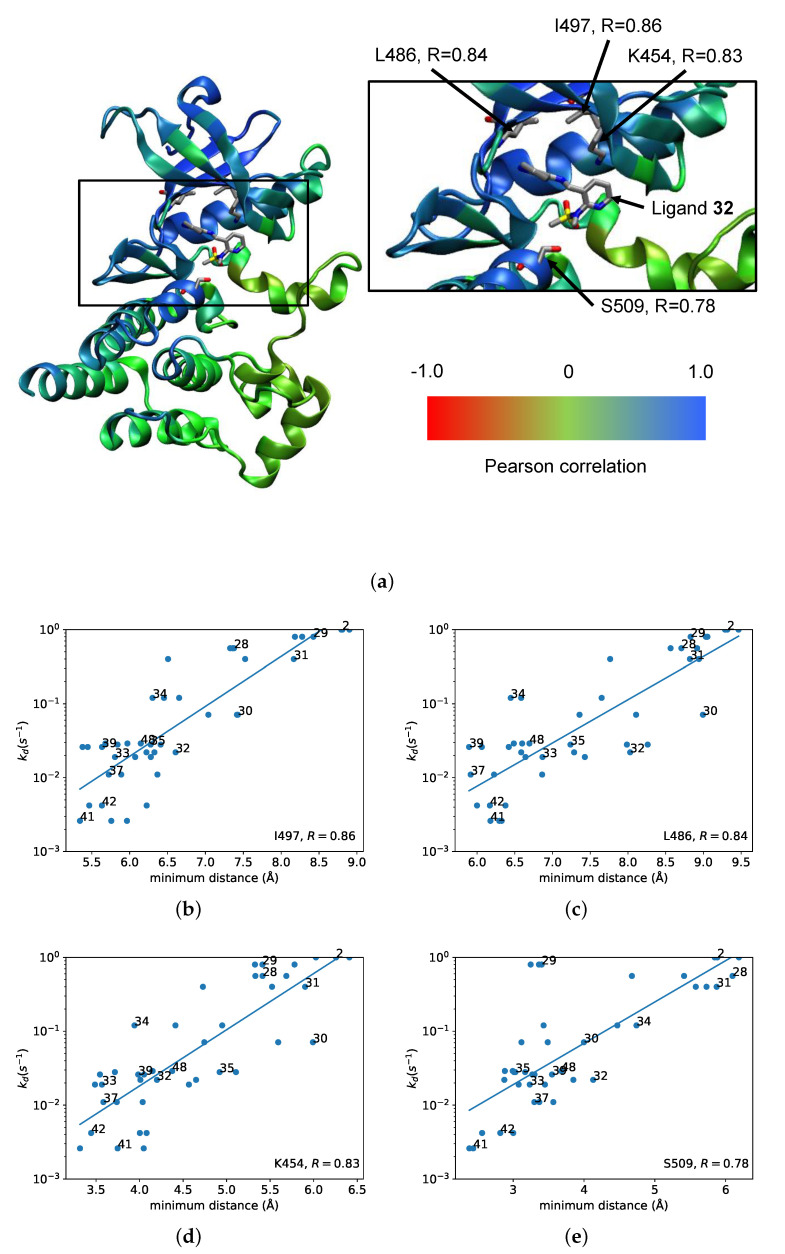
Correlation between experimental dissociation rate and minimum distance between ligand and individual residues. (**a**) The backbone of FAK, color-coded according to the correlation between experimental dissociation rate and minimum distance for each residue. Inset shows the positions of residues Ile 497, Leu 486, Lys 454, and Ser 509 and the bound conformation of ligand **32** for reference. (**b**–**e**) Correlation plots of experimental dissociation rate vs. minimum distance for selected individual residues. (**b**) Ile 497; (**c**) Leu 486; (**d**) Lys 454; (**e**) Ser 509.

In order to study whether contact between the ligand and particular residues could be used to predict dissociation rate, further regressions, also fitted using Equation (3), were conducted in which the experimental dissociation rate was correlated against the minimum distance between the ligand and each residue of the protein. These regressions were done with data points from each of the 42 simulations. Figure 5a shows the backbone of FAK colored by the magnitude of the correlation observed in each regression. Of the 273 residues in the protein, 114 showed positive correlations greater than 0.5. Four residues (Ile 497, Leu 486, Lys 454, and Ser 509) were chosen based on the magnitude of the correlation, their distance from the ligand binding site, and their position in the sequence. Figure 5b–e show the regressions for these individual residues. The highest correlation of 0.86 was observed for Ile 497.

## 3. Exit Pathways for Ligands in SMD Simulations

**Figure 6:**
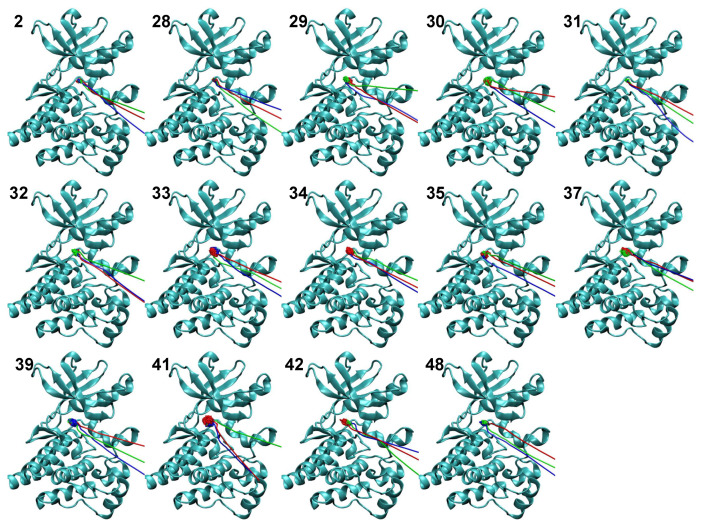
Exit pathways for each ligand in SMD simulations with a force of 400 pN. The pathway taken by the center of mass of each ligand is shown, along with a cartoon representation of FAK for reference. The three exit pathways are shown in three different colors (red, blue and green) for the three independent SMD simulations.

Figure 6 illustrates the pathway taken by the center of mass of the ligand in each of the SMD simulations with a force of 400 pN. For most ligands, the exit pathways obtained from the three simulations are similar near the binding pocket, but may diverge when the ligands reach the surface of the protein. Ligands **30**, **35**, **39**, and **48** showed larger variations even near the binding pocket, but the variations were not substantial.

## 4. Discussion

### 4.1. Low Barrier in Free Energy Simulations

A free energy simulation was carried out using umbrella sampling to identify the location and magnitude of the barrier to exit for three of the ligands (**32**, **2** and **41**). For **32**, this simulation gave an estimate of the barrier height that corresponds to a dissociation rate seven orders of magnitude larger than the experimental rate. There are a number of possible reasons for this discrepancy. The transmission coefficient κ in Eyring’s equation might be much less than 1, although not likely to change the results by more than several orders of magnitude.

The umbrella sampling simulations also exaggerated the differences in binding affinity and dissociation rate of ligand **2** compared to **32**, and produced a barrier height for **41** lower than that for **32**, despite the former ligand dissocating more slowly. This further demonstrates that, despite its computational cost, umbrella sampling simulation has its own limitations in quantitatively predicting dissociation rates. Nevertheless, umbrella sampling was able to broadly classify the ligands of very high (ligand 32 and ligand 41) and very low (ligand 2) residence times.

The SMD simulations also produced varied trajectories for ligand exit for many of the bound ligands. While an effort was made to use long trajectories as a basis for the umbrella sampling simulations in order to base them on the most realistic exit trajectory possible, this variation may have an influence on the calculated barrier heights. Previous simulations have similarly identified multiple pathways for the exit of benzene from the binding site of the T4 lysozyme L99A mutant [52]. This problem is not limited to SMD simulations but also to approaches that use reaction coordinates specified by structural parameters.

As Markov state analysis and the milestoning method also gave comparable dissociation rates as the umbrella simulations, perhaps the most probable explanation for the deviations from experiment would be an error in the force fields. While the CHARMM36 force field for proteins is extensively tested, the CGenFF force field relies on algorithms to choose from a large database of parameters and guess which ones are most chemically appropriate. The CGenFF force field generator also provides a “penalty score” for each parameter, indicating the level of confidence it has in that parameter. Several dihedral parameters for rotatable bonds had high penalty scores, indicating that the CGenFF force field generator had little confidence in the chemical analogy between the parameters in its database and in the molecule. These included the bonds connecting the sulfonamide to the pyridine ring, linking the pyridine ring to the fused pyrrolo-pyridine ring, and linking the pyrrolo-pyridine ring to the benzene ring. Incorrect parameters for these bonds could possibly affect the conformational behavior of **32**.

### 4.2. Choice of Solvation Model for SMD Simulations

Since it was found that simulations were not able to accurately predict the absolute dissociation rate for ligand **32**, we sought to determine whether dissociation rates could be predicted in a more qualitative fashion using more approximate, less expensive computational techniques. In particular, a strong correlation between simulated exit time in SMD simulations and experimental dissociation rates was observed for 14 ligands, confirming the result previously obtained for three of the ligands [44]. This result was obtained despite the use of a relatively approximate simulation setup. Steered molecular dynamics simulation started with only three initial conditions, and an implicit solvent model was used instead of explicit solvent. The FACTS implicit solvent model is a generalized Born model which provides an approximate description of solvation and hydrophobic effects. With the settings used in this work, it can estimate the electrostatic solvation energy of protein conformations to within approximately 3% compared to numerical solutions of the Poisson-Boltmann equation [51]. Although the use of explicit solvent coupled with the particle mesh Ewald method is generally considered to be a superior method of treating these effects, the use of explicit solvent in this instance can also result in frictional and hydrodynamic effects that would not be realistic given the accelerated timescale for ligand exit in the simulations. The FACTS model is also more computationally efficient than previous generalized Born models because it makes use of geometrical calculations rather than numerical integration to calculate the Born radii, supporting our goal of developing a model for qualitative prediction of ligand dissociation rates with minimal computational cost.

### 4.3. Outlier Nature of Ligand ***29***

One of the compounds, ligand **29**, appeared to be an outlier in this regression, with a significantly longer simulated exit time than would be expected based on the correlation involving the other ligands. It is not clear why this is the case. The only ligand for which a bound crystal structure was available was ligand **32**, so in creating starting structures for the simulations, it was assumed that all the ligands bound in a similar way. If this is not the case for ligand **29**, then this ligand could be encountering different free energy barriers to exit from the binding site compared to the other ligands, possibly explaining the outlier nature of the simulated exit times for this ligand.

### 4.4. Forces Influencing the Experimental Dissociation Rates

In order to determine which forces are responsible for the wide range of experimental dissociation rates among the studied ligands, we studied the correlation between the experimental dissociation rate and average values of the total interaction energy and its components. There is a positive correlation between the experimental dissociation rate and the mean value of the total interaction energy, indicating that stronger interactions (with more negative interaction energies) result in slower dissociation. All of the components of the interaction energy also showed statistically significant correlation, except for the electrostatic component. The polar and nonpolar solvation components show the strongest correlations, suggesting a significant role for solvation effects in determining the dissociation rates.

Regressions involving structural features of the protein-ligand complex were also carried out in order to further confirm the type of forces involved and to determine if these quantities could be used to create better models for predicting the dissociation rate. Figure 4 shows regressions for the ligand surface area and total buried surface area. For both surface areas, there is a negative correlation with the dissociation rate, indicating that ligands that are more deeply buried in the binding site dissociate more slowly.

We also studied the correlation of experimental dissociation rate with the minimum distance between the ligand and specific residues of the protein, in order to identify the residues whose interaction with the ligand contributed the most to the differences in dissociation rates. The highest correlations were observed with the minimum distance between the ligand and specific residues of the protein. However, it is to be expected that residues that are nearby in the protein will also show similar correlations, so not all of these correlations represent residue-ligand interactions with a direct effect on the dissociation rate. In order to narrow down these interactions, additional criteria were applied. Only residues that had a minimum distance to the ligand of less than 6 Å were considered further. Of these residues, those with the highest correlations and not adjacent in sequence were chosen for further investigation. This analysis pointed to four residues that had the highest correlation: Ile 497, Leu 486, Lys 454, and Ser 509. Of these residues, two (Ile 497 and Leu 486) have hydrophobic side chains. Ile 453 actually has a slightly higher correlation than Lys 454, but the side chain of Ile 453 faces away from the ligand, while the hydrocarbon part of the Lys 454 side chain comes toward the ligand. Ser 509 was previously identified in the umbrella sampling simulations as being near the transition state, possibly forming some steric hindrance to ligand exit. Hydrogen bonding did not appear to play a significant role in the interactions between these residues and the ligand. A hydrogen bond analysis of the SMD trajectories did not reveal any significant hydrogen bonds between the protein and ligands other than those between the pyrrolopyrimidine ring and Cys 502 that are present in the crystal structure of ligand **32** bound to FAK [45].

## 5. Materials and Methods

### 5.1. Ligands, Initial Structures and Force Fields

**Figure 7:**
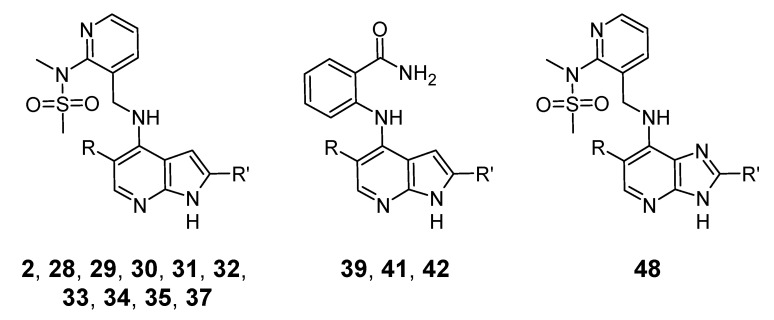
Markush structures of FAK ligands simulated in this work. R groups are shown in Table 3.

Simulations were performed of FAK in complex with compounds **2**, **28**, **29**, **30**, **31**, **32**, **33**, **34**, **35**, **37**, **39**, **41**, **42**, and **48** from the work of Heinrich et al., [45], based on the crystal structure of FAK in complex with compound 32 (PDB code 4GU6) presented in that same work. The structures of the compounds are shown in Figure 7 and Table 3. The FAK protein was modeled using the CHARMM22 force field [53] while the ligands were modeled using the CHARMM36 CGenFF force field [54]. (The CHARMM36 force field for proteins could not be used because it is incompatible with the FACTS implicit solvent model [51] that was used for this work.) An initial reference structure of FAK in complex with ligand **32** was prepared based on the crystal structure of this system from the work of Heinrich et al. [45] (PDB code 4GU6). Hydrogen atoms were added and tautomeric states for histidine assigned using the CHARMM-GUI web server [55].

Each ligand was constructed in Schrodinger Maestro [56] from the initial reference structure, ensuring consistent atom names, and saved in mol2 format. Force fields for each of these ligands were constructed from the mol2 files using the CGenFF web server [54].

### 5.2. Steered Molecular Dynamics Simulations

We conducted steered molecular dynamics (SMD) simulations of the exit of the various ligands of FAK in order to develop models that could be used to predict the dissociation rate. These simulations used an implicit solvent model in order to limit the computational cost. CHARMM [57] was used for initial minimization and heating and the SMD simulations, while NAMD [58,59] was used for the umbrella sampling simulations in explicit solvent described below. The FACTS implicit solvent model [51] was used together with the CMAP corrections originally developed for the GBSW implicit solvent model [60] and also recommended for FACTS. A switching function from 10 to 12 Å was used for the van der Waals interaction as recommended in the FACTS documentation. A hydrophobic surface tension coefficient of 0.015 kcal/mol ${Å}^{2}$ and Debye-Huckel correction corresponding to an ionic strength of 0.15 M were used.

Using this energy function, the initial structure of FAK in complex with each ligand was minimized under harmonic restraints. Each system was then heated to 300 K over 1.5 ns with harmonic restraints of 1.0 kcal/mol ${Å}^{2}$ on each non-hydrogen atom in the system, and equilibrated while relaxing the restraints over a subsequent 1 ns of equilibration. For each system, three steered molecular dynamics simulations [35,36] were then undertaken, starting with the heated and equilibrated configuration and reassigning the velocities in order to ensure independence of the simulations. A choice of four possible applied forces (350 pN, 400 pN, 450 pN, or 500 pN) were used, applied in such a way as to push the N5 atom of the indole or benzimidazole ring away from the amide nitrogen of Cys 502. This choice is based on previous SMD simulations and has been found to give the lowest dissociation barrier and therefore contribute most significantly to dissociation rate [44]. A 2 fs step size was used together with SHAKE to constrain all bonds involving hydrogen. The temperature was maintained at 300 K using Langevin dynamics [61] with a low friction coefficient of 0.1 ${\mathrm{ps}}^{-1}$. Coordinates were recorded every 100 fs. The simulations were continued until the distance between these two atoms exceeded 100 Å, at which point the ligand was deemed to have exited the binding site, the simulation was terminated and the time noted as exit time. This condition was chosen to be sure that the ligand had indeed come out of the protein. From the potential of mean force shown in Results, the ligand can be considered outside at about 20 Å. The difference between 20 Å and 100 Å might appear large but the time to travel this distance is insignificant in comparison to the time spent inside the protein and thus has negligible contribution to the total exit time.

### 5.3. Structural and Energetic Analysis of the SMD Simulations

In order to identify structural features that could be used to predict the experimental dissociation rates of each compound, the SMD trajectories were analyzed using CHARMM, VMD [62] and MDAnalysis [63,64]. MDAnalysis was used to compute the minimum distance between the heavy atoms of each ligand and of each amino acid residue for each frame in each SMD trajectory. From this, the minimum distance of the ligand from each residue was determined over the whole trajectory for each simulation. VMD was used to compute solvent-accessible surface areas of the ligand, the protein, and the entire system, each in its own context, as well as the solvent-accessible surface area of the ligand in the context of the whole system. The buried surface area was determined as the sum of protein and ligand surface areas, each in its own context, less the total surface area of the system. CHARMM was used to compute the interaction energy of the ligand and protein for each frame, which was then decomposed into van der Waals, electrostatic, polar solvation and nonpolar solvation components according to the FACTS implicit solvent model. Regression analysis was then used to determine the correlation of each of these quantities with the experimental dissociation rate, as discussed further in the Results section.

### 5.4. Umbrella Sampling Simulations and Free Energy Surfaces

In order to characterize the free energy surface for the exit of ligands **32**, **2** and **41** from the FAK binding site, umbrella sampling simulations [40] of the three ligands in complex with FAK were carried out. These simulations were based on exit pathways from initial SMD simulations carried out using low forces in the expectation that these pathways would be more likely to be representative of the actual exit pathway. In the case of ligand **32**, the umbrella sampling simulation was based on a separate SMD simulation with a force of 250 pN that had been carried out prior to the group of SMD simulations described above. In the case of ligands **2** and **41**, the umbrella sampling simulations were based on the longest SMD exit simulations with a force of 350 pN taken from the main group of SMD simulations. In each case, windows for umbrella sampling were identified by first RMSD aligning the SMD trajectory with the reference structure along the peptide backbone, then choosing frames in which the center of mass of the ligand was 1 Å from the center of mass in any previous window. Each such center of mass was then used as the center of a harmonic umbrella potential for one of the windows. 30 windows were identified in this way for ligand **32** and 22 for ligand **2**. In the case of ligand **41**, an initial set of 40 windows was identified in this way, but the umbrella sampling simulation did not adequately sample near one of the free energy barriers, so an additional 6 windows had to be added centered on frames from the SMD simulation.

**Figure 8:**
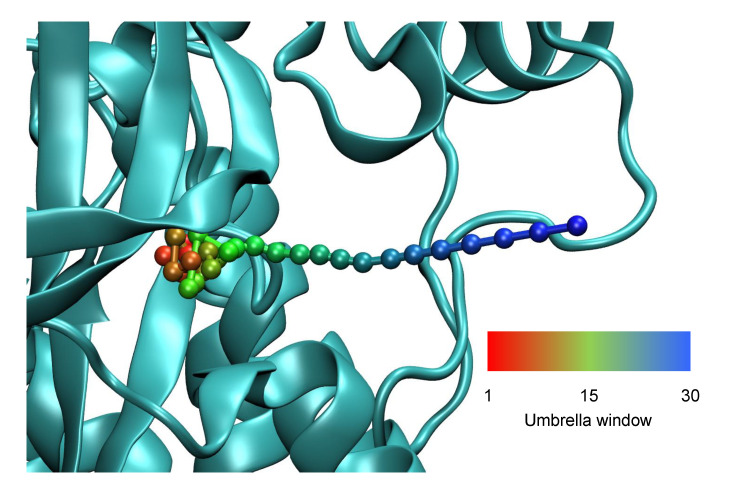
Positions of umbrella potential centers obtained from SMD simulation of ligand **32** exit, superposed on a close up view of the FAK binding site.

For each ligand, the chosen frames from the SMD simulations were each solvated in a rhombic dodecahedral box of TIP3 water [65] and sodium chloride to an ionic strength of 150 mM, and simulated in explicit solvent with the CHARMM 36 force field [66,67] and the particle mesh Ewald method [68]. This simulation consisted of heating to 300 K and equilibration under protocols similar to those used for the SMD simulations, followed by 20 ns of simulation for each window. Langevin dynamics also used to maintain constant temperature, with a damping constant of 10 ps${}^{-1}$. All simulations were also conducted at a constant pressure of 1 atm, with a barostat oscillation time of 100 fs and a decay time of 50 fs. The production simulations used a harmonic umbrella potential centered on the position of the center of mass in the initial frame taken from the SMD simulation and having the following form:

(4) $$U\left(x\right)=U_{\mathrm{FF}}\left(x\right)+\frac{k}{2}\left(r_{\mathrm{CM}}-r_{\mathrm{CM},0}\right)$$

where $U_{\mathrm{FF}}\left(x\right)$ is the potential given by the force field, $r_{\mathrm{CM}}$ is the center of mass of the ligand after alignment of the protein backbone with the initial frame taken from the SMD simulation, and $r_{\mathrm{CM},0}$ was the corresponding position taken from that frame. (These positions are shown in Figure 8.) The force constant *k* of the harmonic restraint was 1.0 kcal/mol ${Å}^{2}$. The use of backbone alignment ensured that overall rotation and translation of the protein had no effect on the umbrella potential.

These simulations were carried out with NAMD [58,59] and the umbrella potential was imposed using the colvars module within NAMD. The first 2 ns of each trajectory was discarded. From the remaining 18 ns of each trajectory, a three-dimensional histogram of the position of the center of mass of the ligand was constructed with a bin size of 0.25 Å in each dimension. These histograms were then combined into a three-dimensional free energy surface for each ligand using the weighted histogram analysis method [69,70]. This method simultaneously corrected for use of the biasing potential and constructed the minimum variance estimate of the free energy surface by combining the histograms from each simulation using the following equations:

(5) $$\begin{array}{ccc} \frac{Z_{i}}{Z_{0}} & = & \int p_{0}\left(r\right)\exp\left[-\beta U_{i}\left(r\right)\right]dr \end{array}$$

(6) $$\begin{array}{ccc} p_{0}\left(r\right) & = & \frac{\sum_{j=1}^{M}h_{j}\left(r\right)}{\sum_{k=1}^{M}\exp\left[-\beta U_{k}\left(r\right)\right]\frac{n_{k}Z_{0}}{Z_{k}}} \end{array}$$

(7) $$\begin{array}{ccc} G(r) & = & -k_{B}T\ln p_{0}\left(r\right) \end{array}$$

where G(r) and $p_{0}(r$ are the free energy surface and probability density in terms of the center of mass r of the ligand, $h_{j}\left(r\right)$ is the histogram obtained from simulation *j*, $U_{j}\left(r\right)=\frac{k}{2}\left(r_{\mathrm{CM}}-r_{\mathrm{CM},j}\right)$ is the biasing potential applied to simulation *j*, and $n_{j}$ is the number of data points for simulation *j*. $\frac{Z_{i}}{Z_{0}}$ is the ratio of the partition function for simulation *i* to that of the unbiased simulation. These equations were solved self-consistently by iterating until the values of $\ln\frac{Z_{i}}{Z_{0}}$ for all simulations *i* had converged to less than 0.001.

Using VMD, each resulting three-dimensional free energy surface was then visualized as contour surfaces together with the protein. This made it possible to determine the location of free energy basins and saddle points corresponding to transition states in relation to the protein. To determine the locations, the isosurface of constant free energy was visualized for increasing free energy levels until two energy basins could connect with each other, as shown in Figure 1a. Each free energy surface was also used to produce a one-dimensional potential of mean force in terms of the distance of the ligand center of mass from its position in the reference structure. This was done by integrating $p_{0}\left(r\right)$, as calculated from Equation (6) over all bins corresponding to a given center of mass distance and converting to a free energy. Due to statistical error in the free energy surface, it is not possible to estimate the transition state free energy with a precision much higher than 0.2–0.3 kcal/mol.

## 6. Conclusions

We have studied the dissociation of 14 ligands from focal adhesion kinase using a combination of umbrella sampling free energy simulations and steered molecular dynamics simulations. While a free energy simulation of three of the ligands showed barrier heights too low to be consistent with the experimental dissociation rate, exit times obtained with steered molecular dynamics simulations showed a strong correlation, making qualitative comparisons possible. There were also strong correlations with most of the components of the interaction energy, particularly the nonpolar component, and with distances to several nonpolar residues. Regression models were also developed that may prove helpful in predicting dissociation rates for other FAK ligands and designing molecules to have a desired dissociation rate.

## Figures and Tables

**Table 1:** Outlier nature of ligand **29**. Slope and standard error of regression line, Pearson correlation coefficient, and root mean squared deviation of the residuals for fits of Equation (2) with and without ligand **29**. Root mean square errors are given in $\log_{10}$ units of the experimental dissociation rate.

	With 29			Without 29		
**Force (pN)**	**Slope**	**Correlation**	**Residual RMS**	**Slope**	**Correlation**	**Residual RMS**
350	−0.61 ± 0.30	−0.50	0.91	−0.89 ± 0.28	−0.70	0.76
400	−0.73 ± 0.21	−0.71	0.62	−0.92 ± 0.19	−0.82	0.52
450	−0.86 ± 0.21	−0.76	0.63	−1.06 ± 0.19	−0.86	0.51
500	−0.54 ± 0.23	−0.56	0.68	−0.78 ± 0.19	−0.77	0.53

**Table 2:** Correlation between experimental dissociation rate and components of the interaction energy and surface area measurements. Slope, standard error, Pearson correlation coefficient, RMS of residuals and *p*-value (for a two-sided test of the null hypothesis of zero slope) for fits of Equation (3) against the mean value of the indicated independent variables over all three simulations conducted for each ligand. Energies are measured in kcal/mol and surface areas in ${Å}^{2}$; the residual RMS is in $\log_{10}$ units of the experimental dissociation rate.

Independent Variable	Slope	Correlation	Residual RMS	*p*-Value
total interaction energy	0.0247 ± 0.0061	0.76	0.56	0.002
van der Waals interaction energy	0.1688 ± 0.0521	0.68	0.63	0.007
electrostatic interaction energy	0.0128 ± 0.0603	0.06	0.86	0.835
polar solvation interaction energy	0.0249 ± 0.0069	0.72	0.60	0.003
nonpolar solvation interaction energy	−0.9763 ± 0.2205	−0.79	0.53	0.001
ligand SASA	−0.0144 ± 0.0046	−0.67	0.64	0.008
buried SASA	−0.0082 ± 0.0027	−0.66	0.65	0.011

**Table 3:** R groups for structures of FAK ligands simulated in this work, corresponding to Markush structures shown in Figure 7. Experimental data as measured using surface plasmon resonance taken from Ref. [45]. * The dissociation rate of compound **2** was outside the instrument range; the value shown is the minimum value possible.

Ligand	R	R${}^{\prime }$	Experimental $K_{D}$ (nM)	Experimental Dissociation Rate (s${}^{-1}$)
**2**	−H	−H	4760	1.0 *
**28**	−CH_3_	−H	1700	0.56
**29**	−H	4-fluorophenyl	6380	0.80
**30**	−CH_3_	4-fluorophenyl	554	0.071
**31**	−CN	−H	147	0.40
**32**	−CN	phenyl	44	0.022
**33**	−CN	4-fluorophenyl	603	0.019
**34**	−CN	cyclohexyl	242	0.12
**35**	−CN	4-n-butylphenyl	430	0.028
**37**	−CF_3_	4-fluorophenyl	24	0.011
**39**	−CF_3_	pyridon-5-yl	12	0.026
**41**	−CF_3_	4-morpholin-4-yl-phenyl	35	0.0026
**42**	−CF_3_	6-morpholin-4-yl-pyridin-3-yl	9	0.0042
**48**	−CF_3_	4-fluorophenyl	33	0.029

## Data Availability

Exclude.

## Abbreviations

The following abbreviations are used in this manuscript:

MD	molecular dynamics
PMF	potential of mean force
FAK	focal adhesion kinase
CHARMM	chemistry at Harvard molecular mechanics
SMD	steered molecular dynamics
FACTS	fast analytical continuum treatment of solvation
CGenFF	CHARMM general force field
CMAP	cross-term map
